# Biochemical predictors for metabolic syndrome in preterm infants according to weight ratio

**DOI:** 10.20945/2359-3997000000237

**Published:** 2020-03-30

**Authors:** Claudia Silveira Viera, Grasiely Masotti Scalabrin Barreto, Rita de Cassia Silveira, Hugo Razzini Oliveira, Beatriz Rosana Gonçalves de Oliveira Toso, Milene Sedrez Rover, Sabrina Grassioli, Ana Tereza Bittencourt Guimarães, Sandra Lucinei Balbo

**Affiliations:** 1 Universidade Estadual do Oeste do Paraná Cascavel PR Brasil Universidade Estadual do Oeste do Paraná, Cascavel, PR, Brasil; 2 Hospital Universitário do Oeste do Paraná Cascavel PR Brasil Hospital Universitário do Oeste do Paraná, Cascavel, PR, Brasil; 3 Departamento de Pediatria Universidade Federal do Rio Grande do Sul Porto Alegre RS Brasil Departamento de Pediatria da Universidade Federal do Rio Grande do Sul, Porto Alegre, RS, Brasil; 4 Secretaria Municipal de Saúde de Cascavel PR Brasil Secretaria Municipal de Saúde de Cascavel, PR, Brasil

**Keywords:** Premature infant, low birth weight infant, longitudinal studies, metabolic syndrome

## Abstract

**Objective:**

Prematurity and low birth weight predispose preterm infants to cardiovascular disease in later life. Is the metabolic profile of these children impacted by the relation between birth weight and gestational age (GA)? This study aimed to evaluate whether the relationship between birth weight and GA of preterm infants has a positive correlation with the metabolic profile from birth to the sixth month of corrected age.

**Subjects and methods:**

This is a longitudinal, prospective study with a cohort of 70 preterm and 54 term infants, who were enrolled in the study and shared into two groups: Appropriate for GA (AGA) and Small for GA (SGA), both classified at birth by Fenton and Kim curves. Longitudinal evaluation of anthropometry measures and blood samples of total cholesterol, glucose, triglycerides, and insulin were collected at birth, NICU discharge, and the sixth month of corrected age. Data were analyzed using descriptive and inferential statistical analysis (ANOVA, Fisher test, Shapiro-Wilk, and Cochran test). The effect size was 0.15, power was 0.92, and confidence interval 95%.

**Results:**

No significant statistical differences were observed in relation to biochemical tests between AGA and SGA groups. However, a significant increase in triglyceride results above the reference values for age in the SGA group was observed throughout the follow-up.

**Conclusions:**

Changes observed in the preterm infant metabolic profile show no correlation with adequacy of birth weight. Preterm lipid profile requires continuous evaluation at follow-up, due to the increased cardiovascular risk in later life.

## INTRODUCTION

Low birth weight (LBW) has been associated with childhood morbidity and mortality, which presents direct influence in later life with the development of diseases such as hypertension, dyslipidemia, and type 2 diabetes. The combination of these diseases with alterations in body size, metabolic parameters, and blood pressure characterize metabolic syndrome (MeS) (1). Evidence has shown that prematurity associated with LBW is another factor that predisposes an individual to MeS (2). According to the literature (3,4), the rapid weight gain and increase in body mass index in the first 12 to 18 months of life among LBW infants tends to cause an overweight condition and increased cardiometabolic risk in childhood and adolescence.

In addition to overweight and obesity, prematurity and LBW have been related to MeS components such as insulin resistance, increased blood glucose, cholesterol, triglycerides, and blood pressure changes. Therefore, a child born under this situation is predisposed to chronic health conditions in adult life (5).

Moreover, nutritional impairments in fetal and neonatal life have consequences related to newborn infants’ growth and development processes, exposing them to a higher probability of metabolic diseases in other stages of life. Furthermore, premature infants are likely to develop changes in their long-term health (6). This understanding underlies the fetal origin theory of chronic diseases in adulthood, which supports the theory of fetal metabolic programming. Thus, prenatal stress causes adaptive changes in endocrine and metabolic processes that become permanently programmed and affect adults’ health (7).

In this context, a suboptimal intrauterine environment in the early stages of life causes repercussions on a preterm infant’s growth, predisposing him or her to long-term health problems such as diabetes and MeS. In the 1980s, Barker and Osmond’s study (8) had already suggested that the uterine environment, when associated with fetal and childhood nutrition, is responsible for scheduling cardiovascular disease in adulthood. In addition, a high prevalence of MeS components have been found among very LBW infants at 2 years old (9). A previous study (10) developed in our research group with a sample of 72 preterm infants evaluated the preterm growth and the evolution of their lipid and glycemic profiles. The research data showed that the preterm infants presented linear growth and an upward curve of the lipid profile variables from birth to sixth month of corrected age. Thus, knowing that prematurity has been related to higher risk for obesity and cardiovascular problems throughout their lives, we sought to compare the lipid and glycemic profiles of premature with term infants. Because preterm and LBW infants’ metabolic changes before the first year of life may be considered a literature gap, the lipid and glycemic profiles were compared to verify whether both groups would be similar.

The LBW and preterm infants had postnatal growth restriction in common; therefore, neither group could maintain the fetal growth pattern. Thus, they presented a high risk of metabolic alterations. Although the literature provides evidence, studies that analyze preterm and LBW infants before the first year of life are not enough to elucidate the lack of research about MeS’s components in these groups. Then the question is whether the metabolic profile of preterm infants is impacted by the relation between birth weight and GA.

In this way, the study aimed to evaluate if the relationship between birth weight and GA of preterm infants has a positive correlation with the metabolic profile from birth to the sixth month of corrected age, in comparison to term infants.

## SUBJECTS AND METHODS

A longitudinal, prospective study was conducted on a preterm infant cohort hospitalized in the Neonatal Intensive Care Unit (NICU) at a teaching hospital in Paraná State, Brazil, from May 2015 to December 2016. This preterm infant cohort was compared to a term cohort that was born at the same teaching hospital during the same period. The study was approved by the research ethics committee under process No. 1,134,712. The informed consent form was read and signed by parents or guardians prior to the study.

### Participant selection and description

The preterm infants eligible for the study were born at the hospital, admitted at the NICU, and under treatment for 7 or more days at the unit, and their blood samples were collected upon admission and discharge from the NICU. In addition, participants who attended all appointments at the outpatient follow-up were included. Exclusion criteria were the presence of congenital malformation, inborn metabolism errors or chromosomal anomalies, and death during hospitalization or after hospital discharge.

Thus, 115 preterm infants and their mothers were selected for the study. From this sample, 70 completed the follow-up and attended the four visits at the follow-up clinic, as follows: first week after hospital discharge; first and third months after discharge; and at the sixth month of corrected age (CA).

The study’s term cohort was composed of newborns without any comorbidities and who were born between 37 weeks and 41 weeks and 6 days of GA. Children of adolescent mothers or those who had a disease were excluded from the sample. Consequently, 162 term infants and their mothers were enrolled in the sample. Among them, 54 completed the follow-up evaluation at the sixth month of life.

### Data collection

At the NICU, preterm infants’ data were collected at admission, covering information related to the delivery, birth, and anthropometric measures. The outpatient appointment was scheduled for the first week after the preterm and term infants were discharged. However, the preterm infants were followed until the sixth month of CA and term infants until sixth month of life. All data were collected by the responsible researcher at the NICU and maternity unit and were tabulated in Excel for Windows and double checked.

The glucose, triglyceride, cholesterol, and insulin blood samples were collected at two points for the preterm and term infant cohorts: 1) from 24 to 72 hours after birth and 2) at the sixth month of CA for the preterm infants and sixth month of life for the term infants. The medical care team required the blood samples as part of the assistance during the NICU or maternity hospitalization and in the follow-up program. However, the blood samples for use in this research were obtained from the material discharged right after the NICU medical team’s required screening. The exams were collected by venipuncture using a butterfly with an evacuated tube and an adaptor, all processed by the institutional laboratory. The exams were realized by dry chemistry method with 10 mg/dL of test sensibility for the triglyceride, 20 mg/dL for glucose, and 50 mg/dL for cholesterol. The insulin exam was analyzed by electrochemiluminescence method with 0.03 nIU/mL of test sensibility.

For anthropometric evaluation, naked preterm or term infants were weighed (presented in grams) on a digital scale (Filizola^®^) with a sensitivity of 5 g. Length measurements (presented in centimeters) were obtained using an anthropometric aluminum ruler with the infant in dorsal decubitus. Until 40 weeks of CA, to calculate the Z score of weight and length and to establish the relation between birth weight and GA by Fenton and Kim’s (11) neonatal growth curves, were used the Fenton calculator (12). After this period, the WHO/2006 growth curves were used. The curves were obtained using the online WHO Anthro calculator, version 3.2.2, from World Health Organization (13).

Therefore, the infants were grouped according to the classification of birth weight to GA; that is, Large for GA (LGA), Small for GA (SGA), and Adequate for GA (AGA). This classification is able to efficiently display fetal growth, considering LGA when the newborn is above the 90th percentile; SGA when the infant is below the 10th percentile, and AGA between 10th and 90th percentiles.

### Analyses

Descriptive statistics were analyzed to assess whether prematurity and its consequences have an influence on or correlation with the preterm infants’ metabolic changes after hospital discharge (mean and standard error of the mean, SD).

The biochemical variables (e.g., glucose, triglycerides, cholesterol, and insulin) were evaluated at two points: admission and at the sixth month of CA/sixth month of life. From the birth weight to classification, the preterm and term infants were divided into two groups: AGA and SGA. All variables were evaluated between each group (AGA and SGA) according to the GA (term and preterm infants) by repeated measures ANOVA test. Each variable was analyzed for the two mentioned times, followed by the LSD-Fisher test.

The variables were previously evaluated regarding the distribution of the data through the Shapiro-Wilk test, as well as the homogeneity of the variances using the Cochran test. All statistical analyses were performed using the Statistica 7.0 program. The tests presented a 0.92 power with a mean effect size of 0.15 and a type I error of 0.05 (Gpower 3.1).

## RESULTS

### Sample characteristics

The sample initially constituted 115 preterm infants, 70 of whom concluded the follow-up. The LGA sample was lost in the follow-up. Among this group, the gender distribution frequency was similar (F = 44%; M = 56%) in initial and final samples. Sixty percent of preterm infants were born within 32-36 weeks, and 40% weighed between 1,000 and 1,499 g. Eighty-nine percent of preterm infants were classified as AGA, whereas 11% were classified as SGA. Forty-four percent of the sample were hospitalized at the NICU for more than 21 days.

The term infant sample was initially constituted of 162 participants; however, 54 completed the sixth month follow-up. The gender distribution frequency was equivalent (F = 50%; M = 50%). Of this group, 85.18% were considered AGA, 7.41% were considered SGA, and 7.41% were LGA. During the follow-up, among the 54 who completed the process, 50 were AGA and four were SGA.

The comparison of anthropometric characteristics (Table 1) between the preterm sample and term sample from birth to six months showed statistical differences in both groups.

**Table 1:** Comparison of preterm and term characteristics at birth and six months of life

Variables (Mean ± SD)	Birth	Six mnth	p-value	Birth	Six month	p-value
	
	T (n = 163)	T (n = 54)		PT (n = 115)	PT (n = 70)	
Weight (g)	3211.3 ± 437.3	7922.2 ± 848.2	< 0.0001	1711.9 ± 728.2	7074.3 ± 1208.2	< 0.0001
Height (cm)	48.3 ± 2.1	66.7 ± 2.3	< 0.0001	40.1 ± 4.8	65.2 ± 3.4	< 0.0001
Cephalic circunference (cm)	33.7 ± 1.5	43.6 ± 1.1	< 0.0001	29.1 ± 3.2	42.5 ± 1.7	< 0.0001

T: term; PT: preterm; g: grams; cm: centimeters.

### Study variables

**Figure 1:**
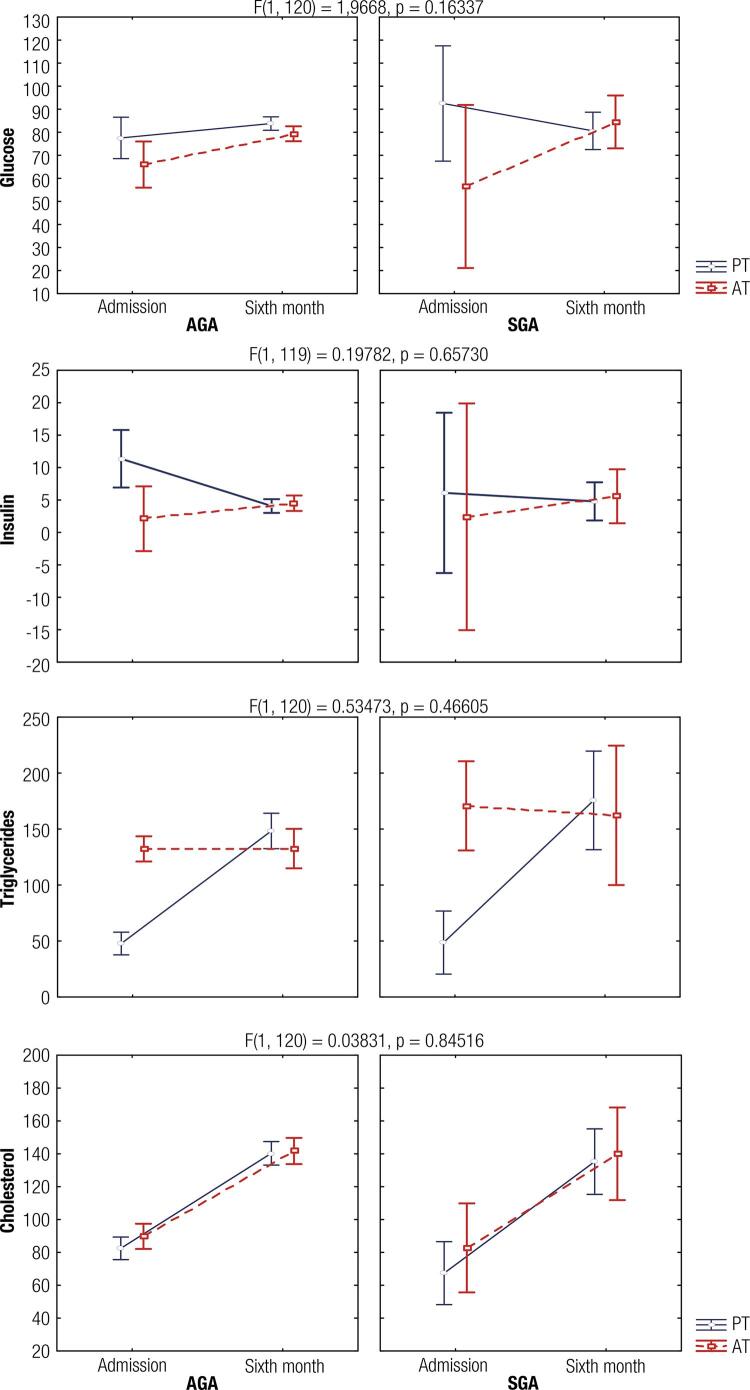
Biochemical variables analyzed in preterm and term infants. AGA = Adequate for GA and SGA = Small for GA. Preterm (n = 70, continuous line). Term (n = 54, dotted line).

During the follow-up, no significant difference in the glucose values (F = 1.97, p = 0.163) was identified in the evaluation of the term and preterm infants, classified as AGA and SGA (Figure 1). However, when assessing only the interaction of GA and follow-up, significant differences (F = 4.05, p = 0.046) were observed. Preterm infants at admission presented significantly higher glycaemia values when compared to term infants, although preterm infants equalized their values to those of term infants during the follow-up.

Regarding the triglyceride variable, the interaction of GA, birth weight classification, and follow-up were verified (Figure 1), nevertheless, they did not present significant statistical differences (F = 0.53, p = 0.466). Nevertheless, the interaction of GA and follow-up showed significant differences (F = 23.93, p < 0.0001), which pointed to preterm infants presenting lower values at admission than those born at term, regardless of whether they are classified as AGA or SGA.

The cholesterol variable (Figure 1) did not present significant statistical differences in the interaction among the GA, birth weight classification, and follow-up (F = 0.038; p = 0.845). Term and preterm infants, classified AGA or SGA, presented low cholesterol at admission and raised their values at the sixth month (F = 94.62, p < 0.0001).

Finally, the insulin variable neither presented statistical differences in the interaction of those factors (F = 0.08, p = 0.771) nor were differences observed in any of the isolated factors.

Metabolic plasmatic parameters showed different profiles during growth. Thus, while the plasma lipid levels (triglycerides and cholesterol) increased during growth, the plasma glucose and insulin levels decreased, suggesting preterm infants altered their metabolism along their growth (Table 2).

**Table 2:** Biochemical parameter predictors of metabolic syndrome in preterm infants compared with term infants. p value by ANOVA for repeated measures

Variables	Preterm (N = 70)		Term (N = 54)		p value^$^
	
	Mean (SD)	Min-Max	Mean (SD)	Min-Max	
**Glycaemia (mg/L)**					
Admission	79.3 ± 44.0	20-285	65.3 ± 20.2	31-121	0.046
Sixth month	83.4 ± 12.6	63-132	79.7 ± 10.0	57-110	
**Triglycerides (mg/dL)**					
Admission	47.8 ± 31.3	13-139	135 ± 50.0	39-242	< 0.0001
Sixth month	151.4 ±70.8	46-338	134.8 ± 50.9	56-273	
**Total cholesterol (mg/dL)**					
Admission	80.7 ± 32.1	25-192	89.2 ± 19.7	25-136	0.495
Sixth month	139.7 ± 29.2	83-240	141.6 ± 27.0	102-231	
**Insulin (uUI/mL)**					
Admission	10.8 ± 23.2	0.5-177.3	2.1 ± 1.9	0.36-11.0	0.229
Sixth month	4.2 ± 4.3	0.19-18.7	4.6 ± 4.0	0.4-20.3	

^$^ p-value of the interaction among preterm and term groups and the follow-up period.

## DISCUSSION

No statistically significant difference was observed in the metabolic profile variables between preterm and term infants, considering the AGA and SGA birth weight classifications. In both groups, the glycemic profile showed stabilization of the serum levels throughout the evaluations. Therefore, insulin presented a decrease until the sixth month of CA for preterm and at the sixth month of life for term infants. Regarding the lipid profile, a gradual increase was noted in serum values from birth to the sixth month for both groups.

The glycaemia at birth for the preterm infant group was higher than the term group. Nevertheless, the preterm infants considerably reduced the glycaemia value at the sixth month of CA. At birth, both groups presented glycemic rates of 54 mg/dL, which is above normal levels for neonatal glycaemia (14). The SGA group showed great variability, whereas the SGA preterm group had higher values than the term group. Monitoring glycemic indexes is fundamental throughout the vital cycle. Thus, a greater relevance may be observed in the neonatal period due to cerebral functioning dependence, in 90%, which is originated from the glucose resulting energy (15).

The insulin results highlight an inverse graph, where higher values are found at birth and reduced levels are identified for both groups along the follow-up. The SGA group presented higher serum insulin values at birth, which decreased until the sixth month. Higher plasmatic concentration of catecholamines (epinephrine, norepinephrine, and dopamine) are preserved in preterm infants with acuter answers for epinephrine when compared to term infants. This situation promotes the insulin decrease and, consequently, an increase in the glucagon concentration (16).

Preterm infants with very low birth weight (<1,500 g) have higher levels of plasmatic insulin, suggesting a peripheral insulin resistance (17). In children, values above 2 uIU/mL are considered hyperinsulinemia. Therefore, the first intervention of the neonate organism is the interruption of insulin secretion when plasmatic glucose concentration decreases below the normal postabsorptive mean of 85 mg/dL (18). For this reason, the insulin concentration values of preterm infants began to diminish along the follow-up to maintain stable plasmatic glucose concentration. Thus, a study (19) highlights that SGA birth status is a significant predictor of insulin resistance, followed by the birth weight-height to GA classification. Furthermore, SGA preterm infants are more vulnerable to developing a problem such as MeS in childhood, adolescence, and adulthood (9,19).

A Spanish study (20) also asserted that the decrease in the postnatal growth curve occurs during the first weeks after birth. Furthermore, the study revealed that SGA preterm infants had higher fasting glucose levels and glucose tolerance in oral test, as well as lower HDL cholesterol levels.

The triglyceride results also do not present a significant statistical difference between the AGA and SGA groups (p = 0.4660). Even so, it is important to highlight that at birth the AGA and SGA preterm infant groups showed lower serum triglyceride values than the term infants. In addition, these values presented gradual increases along the follow-up evaluation, with much higher rates than the Brazilian and European Atherosclerosis Society and the European Federation of Clinical Chemistry and Laboratory Medicine consensus, which were established as adequate for age (15,21). This increase is significant for preterm infant clinical evaluation, indicating the necessity of triglyceride monitoring during this group’s follow-up.

Alterations in fetal and neonatal cholesterol metabolism are important to understand not only during infancy, but for an individual’s long-term health because coronary heart disease has been proposed to be linked to abnormal cholesterol metabolism in the fetus and newborn. Our data showed no significant difference between the term and preterm groups (p = 0.495); both of them presented gradually increasing total cholesterol from birth to sixth months regardless of whether they were AGA or SGA. De Jong and cols. (22) found similar results that showed SGA term and preterm infants had the same total cholesterol values at 2 years old (150 mg/dL).

A recent systematic review (23) presented a comparison between adults born preterm and adults born at term. These results confirmed that prematurity is strongly associated with metabolic syndrome components, and consequently preterm infants are exposed to cardiovascular disease risk in adulthood. In this way, independently of whether a preterm infant’s weight and GA are adequate, this group is vulnerable to future cardiovascular problems. Moreover, it may be important to emphasize that the SGA group showed a continuous triglyceride increase superior to the screenings from the AGA group. These results corroborate studies that have highlighted the SGA preterm group is more likely to present cardiovascular alterations, considering the deprivation that occurs in the intrauterine environment. Thus, a fetal organism tends to prioritize the metabolic demands on organs and noble tissues to the detriment of other areas of growth, resulting in functional and structural tissue impairments and changes in the insulin secretion capacity (5,24).

Evidence from the literature converges (20,25) when it suggests that preterm growth velocity and being born SGA are closely connected to a disproportionately faster rate of fat gain than lean tissue deposition. In other words, fat gain accelerates and leads to recovery or catch-up fat. After birth, SGA preterm infants are exposed to environmental factors such as high energy intake, which may result in metabolic imprinting caused by extra uterine stress (20,26). Among the preterm infants in our study, almost all received parenteral nutrition during NICU hospitalization, which could be considered an important factor in nutritional imprinting. However, the respective roles of enteral and parenteral feeding in later body composition are unclear, and recent randomized controlled trials suggest that manipulation of parenteral nutrition has little effect on lean body mass accretion (27).

Moreover, a study (28) has established that obese children and adolescents remain so in adulthood, predicting Mes. Thus, preterm birth and SGA infants could be at high risk for developing obesity during adolescence. Our data emphasize the necessity of paying attention to the clinical-laboratory aspects during preterm infant follow-up and later growth in life to help patients avoid becoming overweight. Therefore, biochemical tests should be considered in preterm growth evaluation to identify patients’ metabolic profiles, mainly for the SGA preterm group. Consequently, early changes may be recognized, which require adjustments to preterm care management to prevent future complications.

Annual biochemical testing is an important routine in preterm infant follow-up. This analysis could contribute to establishing preterm infants’ metabolic profiles, since prematurity and SGA are considered predictive factors for metabolic changes.

The short period of preterm follow-up observation can be considered a limitation of the study. However, despite the brief follow-up (only until the sixth month of CA), the study may identify the need for attention to the metabolic effects of preterm birth and SGA. Although the SGA sample for both term and preterm infants in our study was reduced and therefore points to another limitation of our research, the findings suggest SGA preterm infants should be considered require special consideration in follow-up interventions. So, when planning a specific care program for this group during hospitalization and after NICU discharge, these elements should be taken into account. According to the literature review, preterm birth and very low birth weight are associated risk factors to cardiovascular diseases. Besides this fact, literature has indicated a higher atherogenic lipid profile among adults who were born preterm (29). Our study revealed high triglyceride values among the analyzed preterm infants, which could be recognized as one of the primary clinical outcomes to detect earlier risk of metabolic syndrome. Then, the lipid profile, mainly triglycerides, may be identified as a serum biomarker in preterm infant metabolic syndrome.

When comparing preterm infants with a control group of term infants of the same age and from the same population, the present study’s results are more reliable. However, it should be noted that the results were stratified into subgroups of adequate or small for gestational age. In spite of the proportions within each group represented, the SGA group was closest to the Brazilian rates, which vary from 5.6 to 10.6% of all live births (29). In our study, 7.4% of the 54 term infants and 11.4% of the 70 preterm infants were classified as SGA. The number of SGA patients is small for the analyses presented in the results, so it may be a study limitation.

In conclusion, the metabolic profile from birth to the sixth month of CA was not influenced by the birth weight and GA of the preterm infant. However, the triglyceride exam could be considered an earlier serum biomarker for MeS among preterm infants. Based on our findings, the importance of following the preterm infant metabolic profile may be evidenced, since triglyceride levels might be a possible predictor of higher cardiovascular disease risk during their lives.

Prematurity and the questions involved in the follow-up of preterm infants require further studies, mainly related to metabolic alterations and early catch up and how these aspects influence a child’s health during late childhood, adolescence, and adulthood. Interdisciplinary long-term longitudinal studies on metabolic markers in preterm infants are challenging when evaluating the risk of developing future alterations, such as MeS, which demand complex and diverse knowledge.

## References

[1] Vaag A, Grunnet L, Arora G, Brons C. The thrifty phenotype hypothesis revisited. Diabetologia. 2012;55(8):2085-8. 10.1007/s00125-012-2589-y PMC3390698 22643933

[2] Villela LD, Soares FVM, Abranches AD, Gomes Junior SC, Méio MDBB, Moreira MEL. Antropometria e composição corporal de recém-nascidos pré-termo na idade gestacional e no peso equivalente ao termo. Rev Nutr. 2015;28(6):619-29.

[3] Sipola-Leppänen M, Vääräsmäki M, Tikanmäki M, Matinolli HM, Miettola S, Hovi P, et al. Cardiometabolic risk factors in young adults who were born preterm. Am J Epidemiol. 2015;181(11): 861-73. 10.1093/aje/kwu443 PMC4445394 25947956

[4] Ong KK, Kennedy K, Castañeda-Gutiérrez E, Forsyth S, Godfrey KM, Koletzko B, et al. Postnatal growth in preterm infants and later health outcomes: a systematic review. Acta Paediatr. 2015;104(10):974-86. 10.1111/apa.13128 PMC5054880 26179961

[5] Parkinson JR, Hyde MJ, Gale C, Santhakumaran S, Modi N. Preterm birth and the metabolic syndrome in adult life: a systematic review and meta-analysis. Pediatrics. 2013;131(4):e1240-63. 10.1542/peds.2012-2177 23509172

[6] Wang G, Divall S, Radovick S, Paige D, Ning Y, Chen Z, et al. Preterm birth and random plasma insulin levels at birth and in early childhood. JAMA. 2014;311(6):587-96. 10.1001/jama.2014.1 PMC4392841 24519298

[7] Silva J, Lamounier J, Cremasco G, Silva V. Metabolic programming interference in the development of obesity and its comorbidities. Rev Salus J Health Sci. 2015;1(1):91-9.

[8] Sedaghat K, Zahediasl S, Ghasemi A. Intrauterine programming. Iran J Basic Med Sci. 2015;18(3):212-20. PMC4414985 25945232

[9] de Jong M, Lafeber HN, Cranendonk A, van Weissenbruch MM. Components of the metabolic syndrome in early childhood in very-low-birth-weight infants. Horm Res Paediatr. 2014;81(1):43-9. 10.1159/000355597 24281139

[10] Barreto GMS, Balbo SL, Rover MS, Toso BRGO, Oliveira HR, Viera CS. Growth and biochemical markers of preterm newborns up to six months of corrected age. J Hum Growth Dev. 2018;28(1):18-26.

[11] Fenton TR, Kim JH. A systematic review and meta-analysis to revise the Fenton growth chart for preterm infants. BMC Pediatr. 2013;13:59. 10.1186/1471-2431-13-59 PMC3637477 23601190

[12] Fenton T. Calculadora Fenton. Clinical actual age percentile and z-score calculator. Available from: http://www.ucalgary.ca/fenton/2013chart

[13] World Health Organization. Child Growth Standards. Software Who Anthro, versão 3.2.2. 2011. 2012. Available from: www.who.int/childgrowth/software/en/

[14] Santos ESRS, Jornada Junior ID. Incidência de hipoglicemia aferida com fita em recém-nascidos grandes para a idade gestacional em um hospital de ensino. Rev AMRIGS; 2014;58(2):105-9.

[15] Sociedade Brasileira de Análises Clínicas – SBAC. Consenso Consenso Brasileiro para a Normatização da Determinação Laboratorial do Perﬁl Lipídico. 2016. Available from: http://www.sbac.org.br/acompanhamento-politico/consenso-brasileiro-para-a-normatizacao-da-determinacao-laboratorial-do-per%EF%AC%81l-lipidico/

[16] Mitanchez D. Glucose Regulation in Preterm Newborn Infants. Horm Res. 2007;68(6):265-71. 10.1159/000104174 17587854

[17] Mitanchez-Mokhtari D, Lahlou N, Kieffer F, Magny JF, Roger M, Voyer M. Both relative insulin resistance and defective islet beta-cell processing of proinsulin are responsible for transient hyperglycemia in extremely preterm infants. Pediatrics. 2004;113(3 Pt 1):537-41. 10.1542/peds.113.3.537 14993546

[18] Thornton PS, Stanley CA, De Leon DD, Harris D, Haymond MW, Hussain K, et al. Recommendations from the pediatric endocrine society for evaluation and management of persistent hypoglycemia in neonates, infants, and children. J Pediatr. 2015;167(2):238-45. 10.1016/j.jpeds.2015.03.057 PMC11891912 25957977

[19] Payal V, Jora R, Sharma P, Gupta PK, Gupta M. Premature birth and insulin resistance in infancy: A prospective cohort study. Indian J Endocrinol Metab. 2016;20(4):497-505. 10.4103/2230-8210.183470 PMC4911839 27366716

[20] Ortiz EM, Gil CM, Muñoz VM, Pérez NJ. Alteraciones metabólicas en 337 prepúberes con retraso del crecimiento extrauterino. Anal Pediatr. 2012;77(4):247-53. 10.1016/j.anpedi.2012.02.011 22494944

[21] Nordestgaard BG, Langsted A, Mora S, Kolovou G, Baum H, Bruckert E, et al.; European Atherosclerosis Society (EAS) and the European Federation of Clinical Chemistry and Laboratory Medicine (EFLM) joint consensus initiative. Fasting is not routinely required for determination of a lipid profile: clinical and laboratory implications including flagging at desirable concentration cut-points-a joint consensus statement from the European Atherosclerosis Society and European Federation of Clinical Chemistry and Laboratory Medicine. Eur Heart J. 2016;37(25):1944-58. 10.1093/eurheartj/ehw152 PMC4929379 27122601

[22] de Jong M, Cranendonk A, van Weissenbruch MM. Components of the metabolic syndrome in early childhood in very-low-birth-weight infants and term small and appropriate for gestational age infants. Pediatr Res. 2015;78(4):457-61. 10.1038/pr.2015.118 26086641

[23] Markopoulou P, Papanikolaou E, Analytis A, Zoumakis E, Siahanidou T. Preterm Birth as a Risk Factor for Metabolic Syndrome and Cardiovascular Disease in Adult Life: A Systematic Review and Meta-Analysis. J Pediatr. 2019;210:69-80.e5. 10.1016/j.jpeds.2019.02.041 30992219

[24] Zohdi V, Sutherland MR, Lim K, Gubhaju L, Zimanyi MA, Black MJ. Low birth weight due to intrauterine growth restriction and/or preterm birth: effects on nephron number and long-term renal health. Int J Nephrol. Int J Nephrol. 2012;2012:136942. 10.1155/2012/136942 PMC3434386 22970368

[25] Ruemmele FM, Garnier-Lengliné H. Why are genetics important for nutrition? Lessons from epigenetic research. Ann Nutr Metab. 2012;60 Suppl 3:38-43. 10.1159/000337363 22614817

[26] Sgarbieri VC, Pacheco MTB. Human development: from conception to maturity. Braz J Food Technol. 2017;20:e2016161.

[27] Darmaun D, Lapillonne A, Simeoni U, Picaud JC, Rozé JC, Saliba E, et al.; Committee on Nutrition of the French Society of Pediatrics (CNSFP), and French Society of Neonatology (SFN). Parenteral nutrition for preterm infants: Issues and strategy. Arch Pediatr. 2018;25(4):286-94. 10.1016/j.arcped.2018.02.005 29656825

[28] Barker DJ, Eriksson JG, Forsén T, Osmond C. Fetal origins of adult disease: strength of effects and biological basis. Int J Epidemiol. 2002;31(6):1235-9. 10.1093/ije/31.6.1235 12540728

[29] Pedraza DP. Baixo peso ao nascer no Brasil: revisão sistemática de estudos baseados no sistema de informações sobre nascidos vivos. Rev Atenção Saúde. 2014;12(41):37-50.

